# Tunneling in the Hydrogen-Transfer Reaction from a Vitamin E Analog to an Inclusion Complex of 2,2-Diphenyl-1-picrylhydrazyl Radical with β-Cyclodextrin in an Aqueous Buffer Solution at Ambient Temperature

**DOI:** 10.3390/antiox10121966

**Published:** 2021-12-08

**Authors:** Ikuo Nakanishi, Yoshimi Shoji, Kei Ohkubo, Shunichi Fukuzumi

**Affiliations:** 1 Quantum RedOx Chemistry Group, Institute for Quantum Life Science (iQLS), Quantum Life and Medical Science Directorate, National Institutes for Quantum Science and Technology (QST), Inage-ku, Chiba 263-8555, Japan; shoji.yoshimi@qst.go.jp; 2 Institute for Advanced Co-Creation Studies, Open and Transdisciplinary Research Initiatives, Osaka University, 2-8 Yamada-oka, Suita, Osaka 565-0871, Japan; 3 Department of Chemistry and Nano Science, Ewha Womans University, Seoul 03760, Korea; 4 Faculty of Science and Engineering, Meijo University, Nagoya 468-8502, Japan

**Keywords:** antioxidant, hydrogen transfer, kinetic isotope effect, tunneling

## Abstract

Recently, increasing attention has been paid to quantum mechanical behavior in biology. In this study, we investigated the involvement of quantum mechanical tunneling in the hydrogen-transfer reaction from Trolox, a water-soluble analog of vitamin E (α-tocopherol), to 2,2-diphenyl-1-picrylhydrazyl radical (DPPH^•^) in a phosphate buffer solution (0.05 M, pH 7.0). DPPH^•^ was used as a reactivity model of reactive oxygen species and solubilized in water using β-cyclodextrin (β-CD). The second-order rate constants, *k*_H_ and *k*_D_, in 0.05 M phosphate buffer solutions prepared with H_2_O (pH 7.0) and D_2_O (pD 7.0), respectively, were determined for the reaction between Trolox and DPPH^•^, using a stopped-flow technique at various temperatures (283–303 K). Large kinetic isotope effects (KIE, *k*_H_/*k*_D_) were observed for the hydrogen-transfer reaction from Trolox to the β-CD-solubilized DPPH^•^ in the whole temperature range. The isotopic ratio of the Arrhenius prefactor (*A*_H_/*A*_D_ = 0.003), as well as the isotopic difference in the activation energies (19 kJ mol^−1^), indicated that quantum mechanical tunneling plays a role in the reaction.

## 1. Introduction

**Figure 1:**
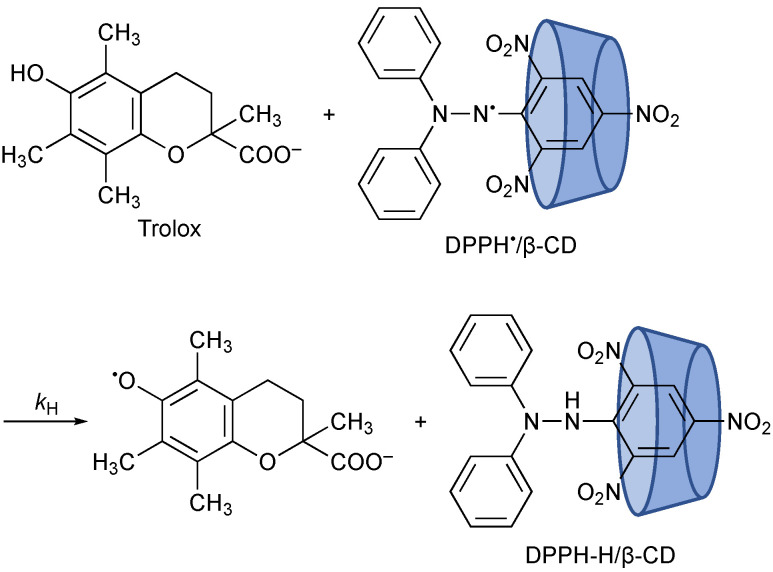
Hydrogen transfer from Trolx to DPPH^•^/β-CD.

Hydrogen-transfer reactions are cornerstones of the radical-scavenging reactions of antioxidants, such as vitamins C (ascorbic acid) and E (α-tocopherol), flavonoids, and so on, where hydrogen atoms (or protons and electrons) are transferred from antioxidants to oxygen radicals as an initial step. On the other hand, increasing attention has been paid to quantum mechanical behavior in biology in recent years [1], such as hydrogen tunneling [2,3,4,5]. Mukai et al. reported a large kinetic isotope effect (KIE, *k*_H_/*k*_D_) of 22.5, observed in the hydrogen-transfer reaction from α-tocopherol to aroxyl radical in ethanol, demonstrating that quantum mechanical tunneling plays a role in this reaction [6]. 2,2-Diphenyl-1-picrylhydrazyl radical (DPPH^•^) is a stable radical and has been used as a reactivity model of reactive oxygen species, to investigate the radical-scavenging reactivity of antioxidants, as well as their mechanism, for more than 60 years [7,8,9]. However, to the best of our knowledge, there has no reports about tunneling in a reaction involving DPPH^•^. Furthermore, the insolubility of DPPH^•^ in water has precluded its use in aqueous solutions, especially in concentrated buffer solutions. We have recently succeeded in solubilizing DPPH^•^ in water by forming an inclusion complex with β-cyclodextrin (β-CD) [10,11]. This enables us to investigate DPPH^•^-scavenging reactivity and the mechanism of antioxidants in aqueous buffer solutions [10,11,12,13]. We report herein the first observation of the temperature dependence of large primary kinetic isotope effects for the reaction of Trolox, a water-soluble analog of α-tocopherol, with β-CD-solubilized DPPH^•^ (DPPH^•^/β-CD) in a phosphate buffer (Figure 1), indicating that quantum mechanical tunneling plays a role in this reaction.

## 2. Materials and Methods

### 2.1. Materials

Trolox and β-CD was commercially obtained from Tokyo Chemical Industry Co., Ltd., Japan. DPPH^•^ and phosphate buffer solution (0.1 M, pH 7.0) were purchased from Fujifilm Wako Pure Chemical Ind. Ltd., Osaka, Japan. D_2_O was commercially obtained from Nacalai Tesque, Inc., Kyoto, Japan. A Milli-Q system (Millipore Direct-Q UV3) (Merck Millipore, Burlington, MA, USA) was used to freshly prepare the water used in this study. DPPH^•^ was solubilized in water by β-CD, according to the procedure described in the literature [10]. The deuterated phosphate buffer solution was prepared by dissolving phosphate buffer powder (Fujifilm Wako Pure Chemical Ind. Ltd., Osaka, Japan) into D_2_O and the pD was adjusted by adding 5 N hydrochloric acid (Fujifilm Wako Pure Chemical Ind. Ltd., Osaka, Japan). The pD values were calculated by adding 0.4 to the corresponding pH values measured using a HORIBA D-51 pH meter (Horiba, Ltd., Kyoto, Japan) [14].

### 2.2. Spectral and Kinetic Measurements

An Agilent 8453 photodiode array spectrophotometer (Agilent Technologies, Santa Clara, CA, USA) was used to record the UV-vis spectra. The scavenging rates of DPPH^•^/β-CD by Trolox in a phosphate buffer solution (0.05 M, pH 7.0) by Trolox were followed by monitoring the absorbance change at 527 nm due to DPPH^•^ (*ε* = 1.1 × 10^4^ M^−1^ cm^−1^) after the mixing of DPPH^•^ in water (Milli-Q) with a phosphate buffer solution (0.1 M, pH 7.0) containing Trolox at a volumetric ratio of 1:1 using a stopped-flow technique on a UNISOKU RSP-1000-02NM spectrophotometer (UNISOKU Co., Ltd., Osaka, Japan), which was thermostated with a Thermo Scientific NESLAB RTE-7 Circulating Bath (Thermo Fisher Scientific, Inc., Waltham, MA, USA). Pseudo-first-order rate constants (*k*_obs_) were obtained by a least-square curve fit, using an Apple MacBook Pro personal computer (Apple Inc., Cupertino, CA, USA). The first-order plots of ln(Abs–Abs_∞_) vs. time (Abs and Abs_∞_ are the absorbance at the reaction time and the final absorbance, respectively) were linear until three or more half-lives, with a correlation coefficient *ρ* > 0.999. In each case, it was confirmed that the *k*_obs_ values derived from at least three independent measurements agreed within an experimental error of ±5%.

## 3. Results and Discussion

**Figure 2:**
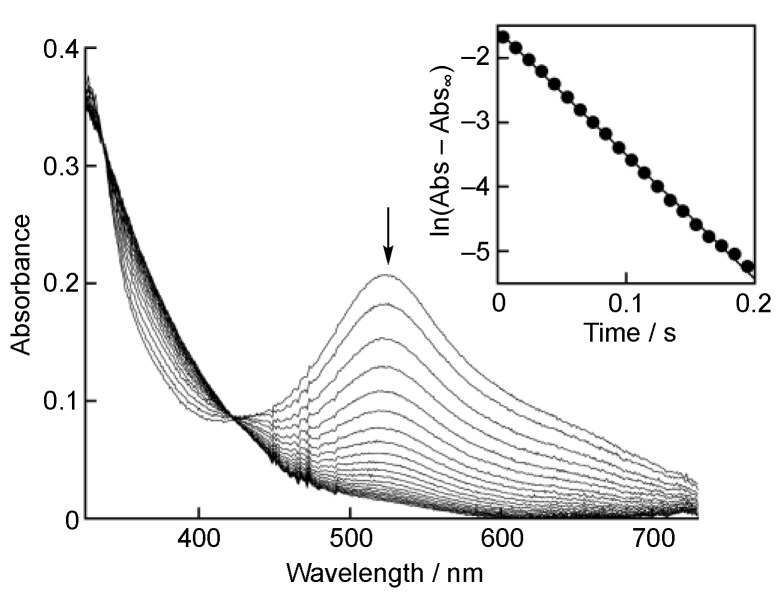
Spectral change (interval: 10 ms) observed during the reaction of Trolox (1.4 × 10^−3^ M) with DPPH^•^/β-CD (1.9 × 10^−5^ M) in phosphate buffer (0.05 M, pH 7.0) at 298 K. Inset: the first-order plot of the absorbance at 527 nm.

**Figure 3:**
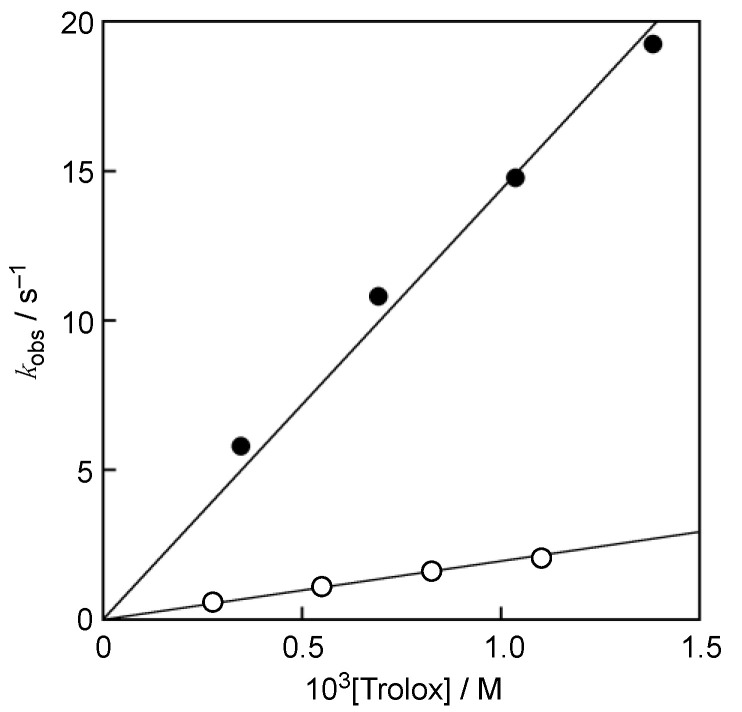
Plots of pseudo-first-order rate constants (*k*_obs_) vs. concentrations of Trolox in phosphate buffer (H_2_O, 0.05 M, pH 7.0) (closed circles) and in phosphate buffer (D_2_O, 0.05 M, pD 7.0) (open circles).

Upon mixing of a phosphate buffer solution (0.1 M, pH 7.0) of Trolox with DPPH^•^/β-CD in water (Milli-Q) at a volumetric ratio of 1:1 on a stopped-flow spectrophotometer, the absorption band at 537 nm due to DPPH^•^ decreased immediately, with clear isosbestic point at 424 nm, as shown in Figure 2. This spectral change indicates that Trolox efficiently scavenged DPPH^•^ in the phosphate buffer. Since the p*K*_a_ value of the carboxylic group of Trolox is known to be 3.89 [15], the carboxylic group was completely deprotonated at pH 7.0 (Figure 1). Thus, the hydrogen transfer occurred from the phenolic OH group in Trolox to DPPH^•^. The decay of the absorbance at 527 nm, which was monitored using a stopped-flow technique, obeyed pseudo-first-order kinetics, when the concentration of Trolox ([Trolox]) was maintained at more than a 10-fold excess of DPPH^•^ concentration (inset of Figure 2). The pseudo-first-order rate constants (*k*_obs_) increased linearly with increasing [Trolox] (Figure 3). The second-order rate constant (*k*_H_) in Equation (1) was obtained from the slope of the plot Equation (2) for the hydrogen transfer from Trolox to DPPH^•^ (Figure 1) in a phosphate buffer solution (0.05 M, pH 7.0) to 1.4 × 10^4^ M^−1^ s^−1^.

When D_2_O was used instead of H_2_O to prepare the phosphate buffer, the phenolic O–H proton in Trolox was replaced by deuteron from D_2_O. The second-order rate constant (*k*_D_) determined for the reaction of Trolox with DPPH^•^/β-CD was much smaller (2.0 × 10^3^ M^−1^ s^−1^) than the *k*_H_ value. Thus, the KIE (*k*_H_/*k*_D_) was calculated to be 7.4, which is slightly smaller than the semi-classical isotope effect for O–H bonds (7.9) [16].

−d[DPPH^•^]/d*t* = *k*_H_[Trolox][DPPH^•^] (1)

*k*_obs_ ([Trolox] > 10[DPPH^•^]) = *k*_H_[Trolox] (2)

The reaction of Trolox with DPPH^•^/β-CD was also carried out in temperature range from 283 to 303 K. Table 1 lists the *k*_H_ and *k*_D_ values determined from the slopes of the linear plots of the *k*_obs_ vs. the Trolox concentrations.

**Figure 4:**
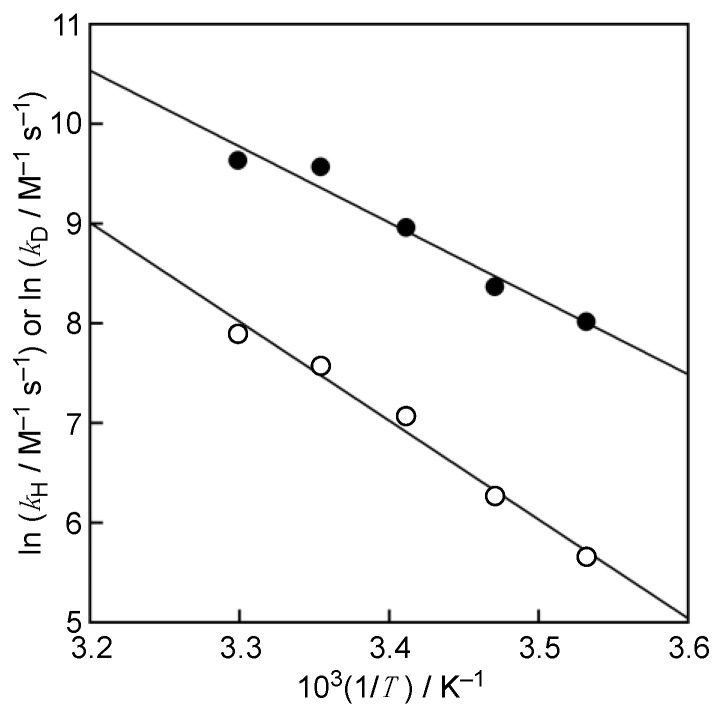
Arrhenius plots of ln *k*_H_ vs. *T*^−1^ (closed circles) and ln *k*_D_ vs. *T*^−1^ (open circles) in phosphate buffer (H_2_O, 0.05 M, pH 7.0) and in phosphate buffer (D_2_O, 0.05 M, pD 7.0), respectively.

Furthermore, as seen in the Arrhenius plots based on the Arrhenius equation Equation (3) (*E*_a_(H), *E*_a_(D): activation energy, *A*_H_, *A*_D_: Arrhenius prefactor, *R*: gas constant and *T*: temperature in K) shown in Figure 4, linear correlations of ln *k*_H_ vs. *T*^−1^ and ln *k*_D_ vs. *T*^−1^ were observed in the reaction of Trolox with DPPH^•^/β-CD in the whole temperature range. From the intercepts and slopes of the linear plots in Figure 4, the Arrhenius prefactors and activation energies were obtained as *A*_H_ = 1.5 × 10^15^ M^−1^ s^−1^, *A*_D_ = 5.2 × 10^17^ M^−1^ s^−1^, *E*_a_(H) = 63 kJ mol^−1^, and *E*_a_(D) = 82 kJ mol^−1^, respectively. The isotopic ratio of ratio, *A*_H_/*A*_D_, was obtained as 0.003, which is beyond the semiclassical limits of 0.4–1.4 [16]. The isotopic difference, *E*_a_(D)–*E*_a_(H), (19 kJ mol^−1^) was significantly greater than the difference in zero-point energies of 5.1 kJ mol^−1^ [16]. These results indicate that quantum mechanical tunneling plays a role in the hydrogen-transfer reaction from Trolox to DPPH^•^/β-CD in a phosphate buffer [17,18,19,20].

ln *k*_H_ = −*E*_a_(H)/(*RT*) + ln *A*_H_ or ln *k*_D_ = −*E*_a_(D)/(*RT*) + ln *A*_D_ (3)

## 4. Conclusions

The solubilization of DPPH^•^ in water by β-CD enabled us to investigate the kinetics of hydrogen-transfer reactions involving DPPH^•^ in aqueous media. The large KIE, as well as the temperature dependence of the KIE observed for the hydrogen-transfer reaction from Trolox to β-CD-solubilized DPPH^•^, indicates that quantum mechanical tunneling played a role in the reaction. To the best of our knowledge, this is the first report about quantum mechanical tunneling in a reaction of DPPH^•^ in aqueous media at ambient temperature.

## Figures and Tables

**Table 1:** *k*_H_, *k*_D_, and *k*_H_/*k*_D_ values for the reaction of Trolox with DPPH^•^/β-CD in phosphate buffer solutions (0.05 M, pH 7.0, or pD 7.0).

*T*/K	*k*_H_/M^−1^ s^−1^	*k*_D_/M^−1^ s^−1^	*k*_H_/*k*_D_
283	3.0 × 10^3^	2.9 × 10^2^	11
288	4.3 × 10^3^	5.3 × 10^2^	8.2
293	7.8 × 10^3^	1.2 × 10^3^	6.6
298	1.4 × 10^4^	2.0 × 10^3^	7.4
303	1.5 × 10^4^	2.7 × 10^3^	5.7

## Data Availability

Data is contained within the article.
